# Orchestrated Regulation of Nogo Receptors, Lotus, AMPA Receptors and BDNF in an ECT Model Suggests Opening and Closure of a Window of Synaptic Plasticity

**DOI:** 10.1371/journal.pone.0078778

**Published:** 2013-11-14

**Authors:** Max Nordgren, Tobias Karlsson, Maria Svensson, Josefin Koczy, Anna Josephson, Lars Olson, Anders Tingström, Stefan Brené

**Affiliations:** 1 Department of Neurobiology, Care Sciences and Society, Karolinska Institutet, Stockholm, Sweden; 2 Department of Neuroscience, Karolinska Institutet, Stockholm, Sweden; 3 Psychiatric Neuromodulation Unit, Wallenberg Neuroscience Center, University Hospital, Lund, Sweden; University of Sydney, Australia

## Abstract

Electroconvulsive therapy (ECT) is an efficient and relatively fast acting treatment for depression. However, one severe side effect of the treatment is retrograde amnesia, which in certain cases can be long-term. The mechanisms behind the antidepressant effect and the amnesia are not well understood. We hypothesized that ECT causes transient downregulation of key molecules needed to stabilize synaptic structure and to prevent Ca2+ influx, and a simultaneous increase in neurotrophic factors, thus providing a short time window of increased structural synaptic plasticity. Here we followed regulation of NgR1, NgR3, LOTUS, BDNF, and AMPA subunits GluR1 and GluR2 flip and flop mRNA levels in hippocampus at 2, 4, 12, 24, and 72 hours after a single episode of induced electroconvulsive seizures (ECS) in rats. NgR1 and LOTUS mRNA levels were transiently downregulated in the dentate gyrus 2, 4, 12 and 4, 12, 24 h after ECS treatment, respectively. GluR2 flip, flop and GluR1 flop were downregulated at 4 h. GluR2 flip remained downregulated at 12 h. In contrast, BDNF, NgR3 and GluR1 flip mRNA levels were upregulated. Thus, ECS treatment induces a transient regulation of factors important for neuronal plasticity. Our data provide correlations between ECS treatment and molecular events compatible with the hypothesis that both effects and side effects of ECT may be caused by structural synaptic rearrangements.

## Introduction

Electroconvulsive therapy (ECT) is used to treat patients with major depressive disorder who do not respond to pharmacologic treatment. However, the treatment does not only attenuate symptoms of depression, it can also cause long lasting memory deficits. Both the amnesia and the superiority of ECT over antidepressant drugs is well-established [1], but the underlying mechanisms of action are still largely unknown. Robust effects on regulation of levels of signaling molecules and trophic factors have been revealed in rats in response to electroconvulsive seizures (ECS), used to model ECT [2]. Many of these effects were observed in hippocampus, an important region for learning and memory [3].

Brain-derived neurotrophic factor (BDNF) is strongly implicated as a factor driving induced plasticity after ECS and is also suggested to be of importance for the antidepressant effects. In support of an antidepressant role of BDNF, serum levels of BDNF have been found to be decreased in patients with major depression [2], [4]. In addition, treatment with ECS increases BDNF protein and mRNA in hippocampus of rat [2], [5], [6].

Long-lasting effects of ECS are likely to also include structural adaptions that affect the state of depression and memory formation [7]. For example, treatment with ECS induces neurogenesis [8], [9] and sprouting of dentate gyrus granule cell mossy fibers [10], events that have been suggested to relate to the clinical efficacy of ECT [11].

The CNS Nogo signaling system inhibits nerve fiber growth. The three ligands Nogo, myelin-associated glycoprotein (MAG) and oligodendrocyte-myelin glycoprotein (OMgp) can all bind to a common receptor, Nogo receptor 1 (NgR1) [12]. Both Nogo and NgR1 are expressed in neurons of the hippocampal formation including the dentate gyrus. Increased neuronal activity causes rapid downregulation of NgR1 [13]–[15]. Lack of NgR1 results in enhanced plasticity in the visual cortex [16]. NgR1 is important for the formation of lasting memories. Thus, the formation of lasting memory is significantly impaired in NgR1 overexpressing mice [17]. Also, levels of the two homologous Nogo receptors, NgR2 and NgR3, are regulated by activity, albeit in the opposite direction [15], [18].

Recently, an endogenous NgR1 antagonist, cartilage acidic protein-1B, which is essential for lateral olfactory tract (LOT) formation, was identified and named LOT usher substance (LOTUS) [19]. When the mouse central nervous system is strongly excited by kainic acid, levels of mRNA encoding the three Nogo receptors and the endogenous NgR1 antagonist LOTUS are altered in the hippocampal formation in a coordinated manner, suggesting allowance of local structural plasticity while maintaining basic network integrity [15].

While alterations of Nogo signaling have been suggested in schizophrenia [20]–[24] and Alzheimer's disease [25]–[29], there has been less focus on depression. Of note however, there are indications that Nogo-B levels might be altered in depressed patients [22]. Accumulating evidence also suggests that glutamate signaling is involved in the pathophysiology of depression. Recently, a randomized double-blind study showed that an ampakine which potentiates the α-amino-3-hydroxy-5-methyl-4-isoxazolepropionic acid receptor (AMPAR), counteracted depression and improved the speed of processing cognitive tasks [30]. Moreover, several studies, both in animals and humans, have shown that acute treatment with the N-methyl-D-aspartate receptor (NMDAR) antagonist ketamine has an antidepressant effect [31], [32]. By blocking the activation of NMDAR [33], ketamine is likely to increase levels of glutamate that can act via other glutamate receptors, including the AMPARs, resulting in a shift from NMDAR to AMPAR activation. The antidepressant effects of ampakines have been suggested to be attributed, at least in part, to neuronal cell proliferation in hippocampus [34], [35]. AMPAR is thought to be composed of two dimers with each dimer comprising two subunits, which typically are of the same subunit type [36]. Four subunits, GluR1-4, have been identified and approximately 70% of AMPARs in hippocampus are composed of GluR1 and GluR2 [37]. Presence of GluR2 renders AMPARs impermeable for Ca^2+^, whereas lack of GluR2 permits Ca^2+^ passage and also increases channel conductance [38]. Interestingly, GluR2, which also has a key role in learning, memory and brain plasticity, is downregulated in hippocampal subregions following kainic acid mediated neuronal excitation, and also after cerebral artery occlusion [39]. However, these responses differ between areas, such that the downregulation of GluR2 is mainly in the pyramidal cell layers, which also are more vulnerable to the cytotoxic effects of the treatments [40]. Additionally, alternative RNA splicing of GluR1 and 2 generates flip or flop forms with different physiological properties and responses when stimulated by glutamate [41].

In this study we analyze to which extent ECS treatment, an animal model of ECT, elicit regulatory responses by genes important for learning, memory and brain plasticity. We hypothesized that induced alteration of NgR1, NgR3, LOTUS, BDNF and AMPA may underlie the antidepressant effects of ECT as well as the treatment-induced amnesia.

## Materials and Methods

### Animals

Adult male Sprague-Dawley rats weighing 200 g were used (n = 55). Animals were housed 3 per cage and kept on a 12 hour light-dark cycle with food and water ad libitum.

### Ethics statement

Experiments were carried out according to guidelines set by the Malmö-Lund Ethical Committee for the use and care of laboratory animals to minimize suffering. All procedures were approved by the Animal Research Ethics Committee of Malmö-Lund (permit no. M69-10).

### ECS

A single bilateral ECS was induced via ear-clip electrodes. Rats were sacrificed by decapitation 2, 4, 12, 24 or 72 hours (n = 7 for each time point) after ECS induction. A group of rats (n = 20) was sham treated and sacrificed 4 hours later, i.e. handled identically to the ECS treated rats but no current was applied. Following decapitation, brains were rapidly removed, frozen on dry ice and stored at −80°C until cryostat sectioning.

Stimulus parameters were 50 mA current, 0.5 s stimulus duration, 10 ms pulse width and 50 Hz unidirectional square wave pulses [8]. Tonic seizure length was defined as the time from the start of the motor seizures until the forelimbs of the rat reached a position perpendicular to the length axis of the body. Clonic movements of the face and forelimbs for a minimum of 20 s, was indicative of limbic motor seizures.

### In situ hybridization

**Figure 1:**
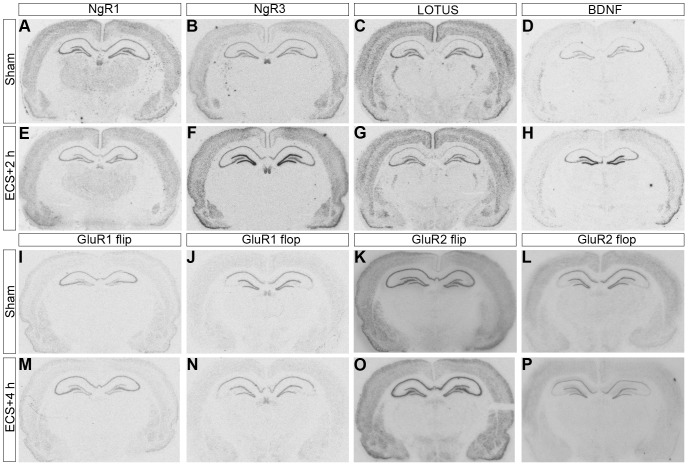
Autoradiograms. Legend: Autoradiograms showing in situ hybridization for NgR1, NgR3, LOTUS, BDNF, GluR1 and GluR2 flip and flop mRNA on coronal brain sections 3.3 mm posterior to bregma on sham and ECS animals. **A–D**. Sham treated rats hybridized with probes detecting NgR1, NgR3, LOTUS and BDNF mRNA, respectively. **E–H**. Rat sacrificed 2 hours after ECS and hybridized with probes detecting NgR1, NgR3, LOTUS and BDNF mRNA, respectively. **I–L**. Sham treated rats hybridized with probes detecting GluR1 and GluR2 flip and flop mRNA, respectively. **M–P**. Rats sacrificed 4 hours after ECS and hybridized with probes detecting GluR1 and GluR2 flip and flop mRNA, respectively. Note changes of mRNA levels in the dentate gyrus.

Frozen tissue blocks were embedded for cryostat sectioning (Tissue-Tek, Sakura Finetek USA Inc., Torrance, CA) and 14-µm sections thawed onto slides (ProbeOn, Fisher Biotech, Pittsburgh, PA). Sectioning was performed approximately 3.3 mm posterior to bregma. High stringency in situ hybridization was performed [42], [43] using ^33^P-labeled oligonucleotide DNA probes complementary to specific sequences of mRNA encoding NgR1 (5′-AGT GCA GCC ACA GGA TGG TGA GAT TCC GGC ATG ACT GGA AGC TGG C-3′), NgR3 (5′-TCA CTG CCA CTC CGT AGT TGA GCT GGG TGG GGT TGC TGT CAT AGT CGG GG-3′), LOTUS (5′-AAG GAC AGC GGC ACT GAG GAG AAG TTG TTG GCC TGG CAG CTC ACG GT -3′), BDNF (5′-CTC CAG AGT CCC ATG GGT CCG CAC AGC TGG GTA GGC CAA GTT GCC TTG-3), GluR1 flip (5′CAA AGC GCT GGT CTT GTC CTT ACT TCC GGA GTC CTT GCT-3′), GluR1 flop (5′CAA AGC GCT GGT CTT GTC CTT GGA GTC ACC TCC CC-3′), GluR2 flip (5′GAG GGC ACT GGT CTT TTC CTT ACT TCC CGA GTC CTT GGC-3′) or GluR2 flop (5′-GAG GGC ACT GGT CTT TTC CTT GGA ATC ACC TCC CCC-3′). Brain sections were compared with appropriate atlases for area identification [44]–[46]. Hybridized sections were air dried for film autoradiography and exposed to film (Kodak Biomax MR film, Kodak, Rochester, New York, USA). Exposure times for NgR1, NgR3, LOTUS, BDNF, GluR1 flip/flop and GluR2 flip/flop were 20, 14, 14, 14, 2, and 3 days, respectively, for optimal quality. Films were developed and scanned using a high resolution scanner (Epson Perfection V750 Pro, Dual lens system, High pass optics; Digital ICE Technologies, Long Beach, CA, USA) for quantification of optical density values (Fig. 1). A ^14^C step standard (Amersham, Biosciences Europe GmbH, Uppsala, Sweden) was included to calibrate optical density readings and to convert measured values into nCi/g. Quantifications were performed using ImageJ [47] by observers blind to grouping. Measurements of optical density of film autoradiograms were performed for NgR1, NgR3, LOTUS, BDNF and GluR1/2 flip/flop in CA1, CA3, CA4 and the dentate gyrus.

Probes were designed not to share significant sequence with any other known rat sequences. We have previously designed and tested several different oligoprobes for each given mRNA of interest. Identical hybridization patterns with 2 or 3 such probes designed to hybridize to different segments of an mRNA of interest, was taken to suggest high specificity, and one probe was then chosen for further experiments. In the present experiments, controls showed expected known patterns of mRNA expression.

### Statistics

Statistical analyses were performed using IBM SPSS Statistics 20. One-way ANOVA followed, when applicable, by a 2-sided Dunnett t post-hoc test for data fulfilling homogeneity of variance. The Games-Howell post-hoc test was used for data not fulfilling homogeneity of variance. Levene's test was used to test for homogeneity of variance. P values of <0.05 were considered statistically significant.

## Results

### NgR1 mRNA expression

**Figure 2:**
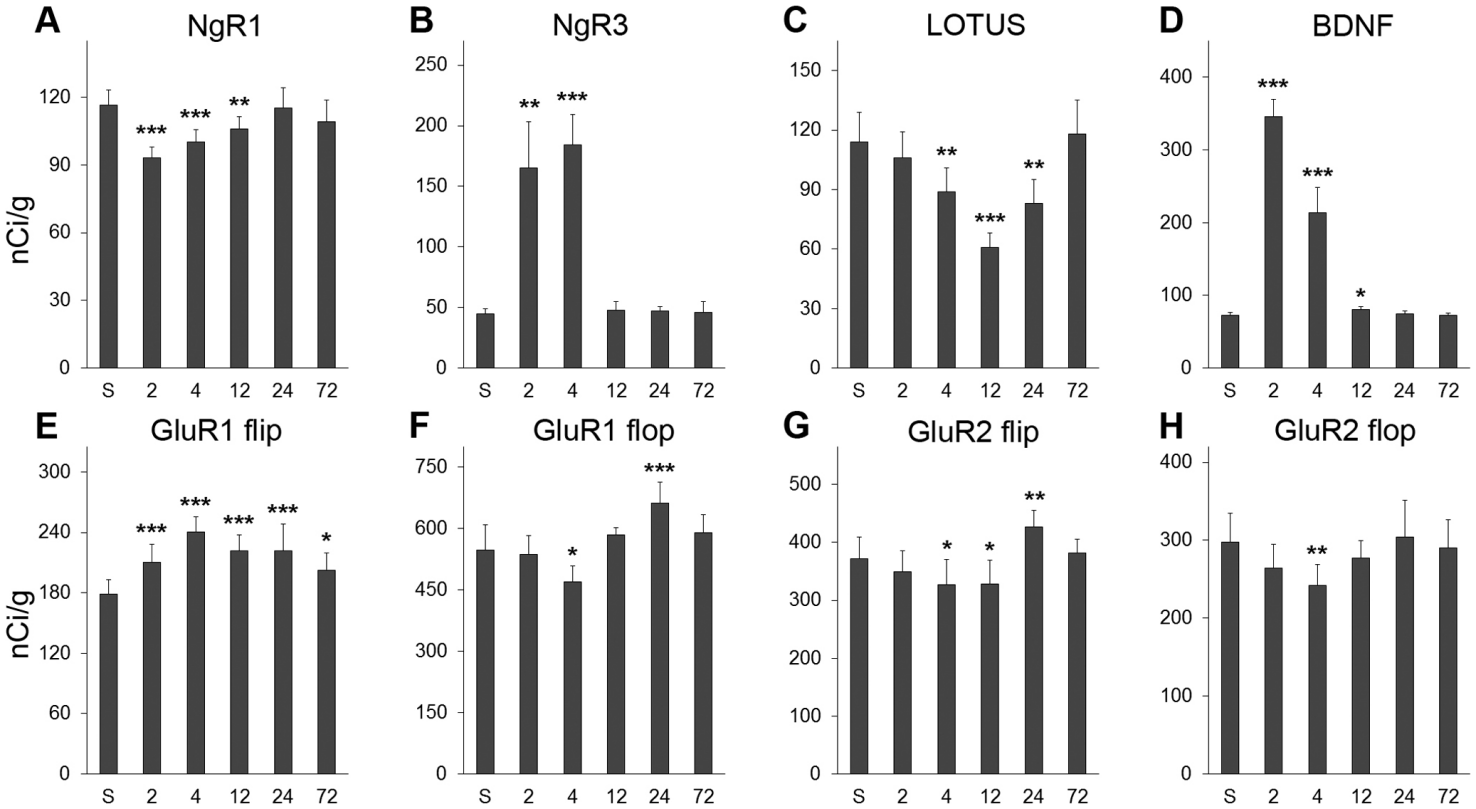
Histograms of results from dentate gyrus. Legend: NgR1, NgR3, LOTUS, BDNF, GluR1 and GluR2 flip and flop mRNA levels expressed as nCi/g in the dentate gyrus of sham (S) and ECS rats. The latter were sacrificed 2, 4, 12, 24 and 72 h after the intervention. **A–H**. NgR1, NgR3, LOTUS, BDNF, GluR1 and GluR2 flip and flop mRNA levels were determined by autoradiography densitometry. Error bars represent +1 SD. Differences from sham treated animals are denoted: * P<0.05, ** P<0.01, *** P<0.001.

A single ECS induction caused a distinct and transient downregulation of NgR1 mRNA levels in the dentate gyrus (Fig. 1 and 2). The reduction was significant at 2 (P<0.0001), 4 (P<0.0001) and 12 h (P<0.01) after ECS as compared to sham treated animals. NgR1 mRNA levels had returned to sham levels 24 hours after ECS. Due to hybridization artifacts, three rats were removed (before slides had been decoded), one each from the groups sham, 2 h and 24 h. There was no significant difference in other investigated hippocampal regions (CA1, CA3 and CA4) (Table 1).

**Table 1:** Mean mRNA levels with standard deviations and significance levels.

Gene		S	2 h	4 h	12 h	24 h	72 h	ANOVA
**NgR1**	**CA1**	93±4	89±6	97±4	92±5	97±8	91±9	0.097
	**CA3**	117±8	113±6	119±11	118±8	123±11	116±11	0.51
	**CA4**	132±8	120±11	135±13	130±10	131±7	131±15	0.15
	**DG**	117±7	93±5***	100±5***	106±6**	115±9	109±10	<0.0001
**NgR3**	**CA1**	37±6	43±9	46±6*	40±6	37±5	38±7	0.027
	**CA3**	46±7	57±11*	69±12***	51±7	46±8	48±10	<0.001
	**CA4**	43±7	59±14	78±13**	52±9	42±5	43±7	<0.001
	**DG**	45±7	165±38**	184±25***	48±7	47±4	46±9	<0.001
**LOTUS**	**CA1**	63±7	66±5	67±11	63±6	71±7	68±8	0.22
	**CA3**	147±21	148±16	152±19	149±14	163±28	152±15	0.62
	**CA4**	113±17	116±18	112±11	98±16	110±28	115±16	0.47
	**DG**	114±15	106±13	89±12**	61±7***	83±12**	118±17	<0.001
**BDNF**	**CA1**	39±6	55±3***	53±5***	40±3	48±8**	43±4	<0.0001
	**CA3**	75±4	85±5***	86±4***	70±3**	72±3	73±4	<0.0001
	**CA4**	72±4	87±4***	82±4***	63±4***	65±5**	68±3	<0.0001
	**DG**	73±4	346±24***	213±35***	80±4*	74±5	73±3	<0.0001
**GluR1 flip**	**CA1**	345±41	362±41	350±43	335±25	342±44	352±34	0.86
	**CA3**	472±54	497±39	485±39	464±28	494±51	502±34	0.46
	**CA4**	434±44	458±29	429±32	416±24	444±44	467±44	0.14
	**DG**	179±15	210±18***	240±16***	221±16***	222±26***	202±18*	<0.0001
**GluR1 flop**	**CA1**	406±50	409±50	396±21	355±11	428±42	427±50	0.18
	**CA3**	111±12	102±15	107±11	109±5	104±6	110±8	0.56
	**CA4**	104±8	104±7	104±11	100±8	102±5	106±12	0.91
	**DG**	548±61	536±46	470±39*	585±19	663±51***	590±44	<0.0001
**GluR2 flip**	**CA1**	385±41	374±55	393±67	373±30	383±20	364±25	0.81
	**CA3**	429±43	440±47	442±57	419±18	456±40	431±22	0.58
	**CA4**	490±61	489±49	487±79	450±39	524±37	486±32	0.27
	**DG**	371±38	349±36	327±44*	329±41*	426±29**	382±24	<0.0001
**GluR2 flop**	**CA1**	235±34	217±32	230±28	220±23	242±37	252±34	0.31
	**CA3**	68±15	71±7	69±10	61±7	62±10	75±16	0.28
	**CA4**	95±19	97±12	87±12	80±10	90±13	97±17	0.21
	**DG**	298±37	264±31	242±26**	278±22	304±48	290±36	0.008

Legend: Mean mRNA levels ± SD expressed as nCi/g for all genes and regions evaluated. Values are rounded to nearest integer. N = 20, 7, 7, 7, 7, 7 for sham (S), 2, 4, 12, 24 and 72 h respectively, except for NgR1 (n = 19, 6, 7, 7, 6, 7) and GluR1 flop (n = 13, 5, 5, 4, 5, 5), due to artifacts. Last column displays P-values for one-way ANOVA for groups (sham, 2, 4, 12, 24 and 72 h) for the current gene and region. Asterisks represent significance between the current group and sham group tested with 2-sided Dunnett t post-hoc test for data fulfilling homogeneity of variance and Games-Howell post-hoc test for data not fulfilling homogeneity of variance.

* P<0.05,

** P<0.01,

*** P<0.001.

**CA** = Cornu Ammonis, **DG** = Dentate gyrus.

### NgR3 mRNA expression

The expression of NgR3 increased in all brain areas analyzed following ECS compared with sham, but was most marked in the dentate gyrus (Fig. 1 and 2) where levels were significantly higher at 2 (p<0.01) and 4 h (p<0.001) before returning to baseline levels. A similar time scale was seen in CA1, CA3 and CA4, in which areas all levels appeared to peak around 4 h and had returned to baseline by 12 h (Table 1).

### LOTUS mRNA expression

**Figure 3:**
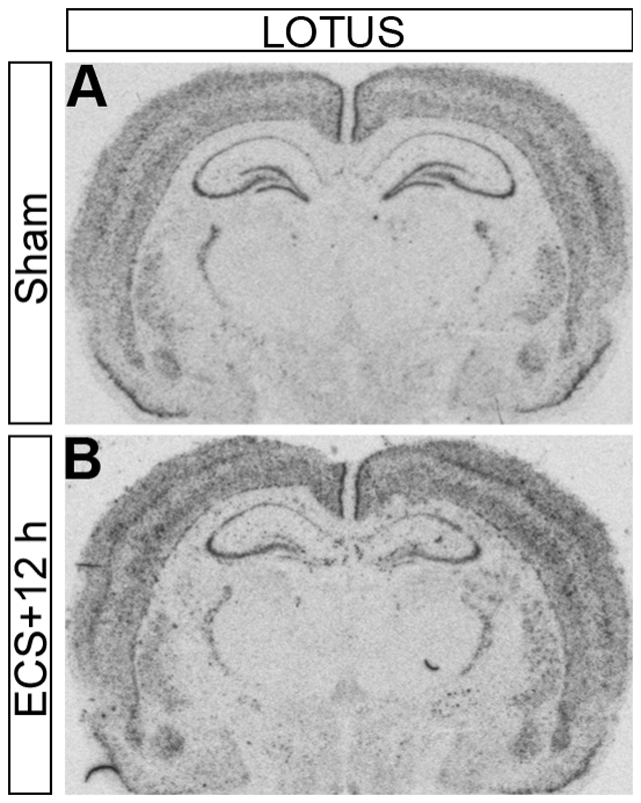
Autoradiograms. Legend: Autoradiograms showing in situ hybridization for LOTUS mRNA on coronal brain sections 3.3 mm posterior to bregma on sham treated and animals sacrificed 12 h after ECS. Note the decreased mRNA signal in the dentate gyrus.in the ECS treated animals.

The level of LOTUS mRNA was significantly downregulated in the dentate gyrus at 4 (Fig. 2) (p<0.01), 12 (p<0.001) (Fig. 2 and 3) and 24 h (p<0.01) following ECS compared with sham, before returning to baseline values at 72 h (Fig. 1, 2 and 3). No change in LOTUS mRNA was detected in other hippocampal subfields after the ECS treatment (Table 1).

### BDNF mRNA expression

The expression of BDNF mRNA in the dentate gyrus was strongly and transiently upregulated (Fig. 1 and 2). Upregulation was observed at 2 (P<0.0001), 4 (P<0.001) and 12 h (P<0.05) after ECS compared with sham. BDNF mRNA in the ECS group had returned to sham levels in the dentate gyrus after 24 h.

For CA1, CA3 and CA4 regions, a small upregulation of BDNF mRNA was noted 2 and 4 h after ECS (Table 1). This upregulation was followed by a small downregulation that reached statistical significance at 12 h in CA3 and at 12 and 24 h in CA4.

### GluR1 and GluR2 flip and flop mRNA expression

GluR1 flip mRNA expression was upregulated in the dentate gyrus by ECS at all time points; 2 h (P<0.001), 4 h (P<0.0001), 12 h (P<0.0001), 24 h (P<0.0001) and 72 h (P<0.05) (Fig. 1 and 2). GluR1 flop mRNA was downregulated in the dentate gyrus 4 h (P<0.05) after ECS. Interestingly, this downregulation was followed by upregulation at 24 h (P<0.001).

GluR2 flip and flop mRNA levels were downregulated in the dentate gyrus 4 h (P<0.05 and P<0.01 respectively) after treatment with ECS compared with sham. GluR2 flip was also downregulated at 12 h (P<0.05), followed by upregulation at 24 h (P<0.01).

No significant difference was seen between groups in CA1, CA3 and CA4 with respect to levels of mRNA encoding GluR1 flip or flop, or GluR2 flip and flop (Table 1).

## Discussion

Understanding of the biological processes underlying the antidepressant effect as well as the amnesia seen in patients after ECT is needed in order to optimize positive effects of treatment, while minimizing negative side effects, and may also help in the search for more potent pharmacological treatments. Here, we report transient regulation of mRNA encoding genes suggested to be important for structural and functional plasticity in a rat ECT model. Our study has limitations. Thus changes in mRNA levels do not necessarily reflect changes in protein levels and functional changes. Nevertheless, our findings suggest possible mechanisms involving the Nogo system, BDNF and AMPA receptors for both the antidepressant and amnesia effects of the intervention. Following a single ECS event, NgR1, suggested to inhibit structural plasticity and to regulate the formation of lasting memories [12], [13], [17], was rapidly downregulated in the dentate gyrus of the hippocampal formation. This downregulation follows a pattern found in several other experimental situations associated with increased plasticity [13]–[15], [25]. Downregulation of NgR1 expression was noted at the same time points after ECS (2 and 4 h) as when NgR3 and BDNF expression was upregulated. This is also similar to the pattern observed in the dentate gyrus in rats and mice injected with the seizure-inducing agent kainic acid for NgR1 [13], [15], NgR3 [15] and BDNF [13] mRNA species. The functional significance of downregulation of NgR1 and upregulation of NgR3 needs to be analyzed in functional assays. Since Nogo and OMgp have high affinity for NgR1, while chondroitin sulfate proteoglycans (CSPGs, ligands for NgR3 [48]) have high affinity for NgR3, our data suggest that the influence of Nogo and OMgp is decreased during a short time window, and in a spatially restricted manner, as dictated by the extracellular matrix.

Interestingly, regulation of mRNA encoding LOTUS, an endogenous antagonist for NgR1, differs significantly when comparing the responses to kainic acid and ECS. Kainic acid induces a robustly increased expression, while ECS leads to a transient decrease of LOTUS mRNA. There are major differences in the biological response to the two interventions. First, the seizures that follow a single kainic acid dose are long lasting and animals may show sporadic seizure activity up to 72 h after treatment [49]. In contrast, the seizures after a 0.5 s ECS event terminate within minutes. This is compatible with the lesser (although strongly significant) magnitude of NgR1 mRNA downregulation found here to be caused by ECS, as compared to the decrease induced by kainic acid [13], [15], [17]. We further note that the effect on LOTUS of ECS is not only opposite to that seen after kainic acid, but also delayed, suggesting a role in effective reversal of an ECS induced state of increased plasticity.

The effects of antidepressant treatments are most likely associated with long term functional and structural modifications of synaptic circuitry in the brain, and the neurotrophic factor BDNF appears to have an important role for the plasticity that occurs after ECS treatment. However, regulation of BDNF is crucial but not sufficient for the occurrence of e.g. the ECS-induced mossy fiber sprouting that may relate to the clinical efficacy of ECT. In support for this, ECS treated BDNF heterozygote knockout mice, had a diminished increase in BDNF levels after treatment, with a corresponding reduction in sprouting of hippocampal mossy fibers compared with wild-type mice [10]. However, direct infusion of BDNF into hippocampus does not induce sprouting of mossy fibers [10]. This suggests that a dynamic regulation of BDNF levels is important for structural plasticity and that ECS treatment targets additional factors that are essential for the initiation of sprouting. One candidate mechanism for the support of sprouting could be the downregulation of NgR1 expression as demonstrated here, since interrupting NgR1 binding to its ligands promotes sprouting [50].

The neuronal basis underlying the memory side effects of ECT is under investigation. However, it has been suggested that memory dysfunction may be caused by alterations in hippocampal synaptic efficacy [51]. Because alterations of Nogo signaling impacts the formation of long-term memories [17], long-term potentiation (LTP) [52], [53] and neuronal sprouting [16], [18], [54], [55], it is possible that NgR1 is involved also in the memory impairments seen in some patients undergoing ECT. Pharmacologically, pretreatment with propofol, which potentiates γ-aminobutyric acid receptor A (GABA_A_ receptor) activity and blocks sodium channels, selective glucocorticoid antagonists and selective non-steroidal anti-inflammatory cyclooxygenase-2 inhibitors, all attenuate the amnesia effect of ECS in animal models [56]–[59]. Specific interaction of Nogo signaling and the described pharmacological agents is currently unknown.

Patients undergoing ECT are routinely oxygenated to prevent possible damage caused by hypoxia. In rats, it has previously been shown that ECS induces proliferation of novel endothelial cells in hippocampus, regardless of whether ECS is administered to individuals that are oxygenated or not [60]. Thus, it is likely that ECT induces novel endothelial cells and vascular growth in patients. In the present study, the animals were not oxygenated during ECS and thus we cannot fully exclude that a hypoxic response might contribute to the observed effects on mRNA levels.

Chronic ECS has been shown to upregulate expression of GluR1 [61]. Here, we studied the expression of GluR1 flip and flop and GluR2 flip and flop mRNA levels at different time points after a single ECS event. The upregulation of the AMPAR subunit GluR1 flip (2–72 h) and the initial downregulation of GluR1 flop (4 h) in the dentate gyrus are noteworthy since a shift from the GluR1 flop to the flip form increases conductance when AMPARs are stimulated by glutamate [41]. Also, the downregulation of both GluR2 flip (4–12 h) and flop (4 h) is interesting, as AMPARs lacking GluR2 subunits permit passage of Ca^2+^ [38], [41]. Association between the Nogo and AMPA system has recently been shown in vitro. Treating hippocampal neurons for 72 h with siRNA specific for NgR1 or Nogo-A, thus reducing NgR1 or Nogo-A protein, both increased GluR1 and GluR2 protein [62].

We hypothesize that ECS opens a transient time window of markedly enhanced structural synaptic plasticity. Decreased levels of NgR1 shortly after ECS, coupled with a strong increase of BDNF and efficient AMPA receptors that are more Ca2+ permeable, are compatible with this hypothesis, and may underlie the therapeutic effects of ECT as well as the negative side effects.

At later time points, LOTUS (4–24 h) is downregulated, possibly, as noted above, to increase inhibition and closing the time window of high plasticity. GluR1 flop and GluR2 flip are upregulated (24 h) and GluR2 flop is normalized (12–72 h). This time-dependent regulation of GluR subunits with early downregulation of GluR2 (4–12 h) together with upregulation of BDNF (2–12 h) following ECS indicate that the window of plasticity with increased neuromodulation is followed by a later phase during which the synaptic network is stabilized in a new, slightly altered configuration. LTP induction has been shown to produce a transient incorporation of GluR2-lacking Ca^2+^-permeable AMPARs in hippocampal neurons, followed by replacement of GluR2-containing AMPARs 25 min after LTP induction [63]. Prolonged Ca2+ entry through AMPARs can cause neural degeneration [64]. Hence the dynamic alteration of synaptic AMPAR subunit types might be necessary to avoid degenerative events during episodes of synaptic plasticity.

## Conclusions

Rats subjected to a single ECS event demonstrate a complex, transient regulation of levels of Nogo receptors, BDNF and AMPA receptors in the dentate gyrus and other parts of the hippocampal formation. It is suggested that these events are temporally and spatially orchestrated to open a time window permissive to synaptic plasticity, followed by closure of this window, leading to a lasting alteration of synaptic circuitry. This could underlie both the beneficial antidepressant effects and the amnesic side effects of ECT treatment.
